# Assessing the utility of ultrasound in Liberia

**DOI:** 10.4103/0974-2700.41785

**Published:** 2008

**Authors:** Simon Kotlyar, Christopher L Moore

**Affiliations:** 1 Section of Emergency Medicine, Yale University School of Medicine, Department of Surgery, New Haven, CT, USA; John F. Kennedy Medical Center, Monrovia, Liberia

**Keywords:** Africa, education, ultrasound

## Abstract

Sub-Saharan Africa has sparse imaging capacity, and data on ultrasound (US) use is limited. We collected prospective data on consecutive patients undergoing US to assess disease spectrum and US utility in Liberia. A total of 102 patients were prospectively enrolled. Average age was 33 years (0-84), 80% were female. US indications were: 53% Obstetrics/Gynecology (OB/GYN) (24% gynecologic, 17% second/third trimester, 12% first trimester), 14% hepatobiliary, 10% intraperitoneal/intrathoracic fluid, 8% cardiac, 5% focused assessment of sonography in trauma, and 4% renal. US changed management in 62% of cases. Greatest impact was in first trimester OB (86%), FAST (83%), ECHO (80%), and second/third trimester OB (77%). US changed management in 47% of right upper quadrant and 33% of gynecologic studies. Curvilinear probe addressed over 80% of need. The primary role for US in developing countries is in management of obstetrics, with a secondary role for traumatic and a-traumatic abdominal processes. Most needs can be met with the curvilinear probe. Training should begin with obstetrics and should be a primary focus for curriculum.

In the majority of sub-Saharan African countries, there is a marked paucity of diagnostic equipment available to the clinician. As such, medical providers are forced to treat and make decisions with limited objective data. More than half the world's population does not have access to at least some form of radiological examination and there is a near absence of trained radiologists in Africa.[1] When considering the available diagnostic options, the technical and financial constraints make computed tomography (CT) practically unavailable, and the World Health Organization (WHO) estimates that about 60% of the world cannot be X-rayed for anything, again often as a result of fiscal and maintenance issues.[2] In light of this crisis, ultrasound (US) technology has the potential to emerge as a cornerstone of diagnostic imaging in developing countries.[2–10] Ultrasonography has been recommended for developing countries by the WHO and specifications for a Basic Radiological System, the WHO-BRS, have been produced.[11] Ultrasound technology is relatively inexpensive, carries no known associated risks, and provides for real-time images and data available to the practitioner.

In the context of these realizations and recommendations, there has been significant interest in the implementation of US technology in resource poor settings. Much has been written about the implications of US and the visions of potential impact; however, few studies have sought to determine the spectrum of disease encountered and the potential benefit that US could provide as an aid to diagnosis and treatment.[2–6,8–10]

As part of an ongoing collaboration between our institution and the tertiary referral hospital of Liberia, we sought out the prospect of introducing US technology to their scope of diagnostic modalities. In the context of this implementation, we collected prospective data to asses the spectrum of disease and the utility of US in diagnosis and treatment of inpatient disease processes. In addition, we planned to make an assessment of the impact that US had on patient treatment and hospital course for each specific disease process.

## PATIENTS AND METHODS

### Site

The study was carried out at John F. Kennedy (JFK) Medical Center in the capital city of Monrovia, Republic of Liberia. JFK Hospital is the government tertiary referral hospital for the country (200 beds) and was disable after years of civil war and military conflict. As such, the capacity of the institution has been severely impaired, with limited physical, technical, and human resources. The hospital provides basic surgical, medical, obstetric and pediatric services, and has sparse pharmacy and lab capacities. Electrical power is available but unreliable, and pluming and running water have just recently been restored. At the time of study, plain radiography was readily available. CT was not available in Liberia and US studies were only provided in select private sector hospitals.

### Patients

Data were collected with the support of local administrative bodies and approval institutional review board (Yale University Human Investigations Committee). All examinations were free of charge and were performed as part of routine patient care. Ultrasonography was performed by one of two emergency physicians with residency training in focused emergency US that met or exceeded the American College of Emergency Physicians (ACEP) recommendations for credentialing in emergency US.[12]

Prospective data were collected during a 5-week period in October-November of 2006. A total of 102 consecutive patients who received US were enrolled during this time period. Ultrasonographic studies were performed on inpatients in the medical, pediatric, surgical, and obstetrical wards upon request of the treating physician. Studies were available 6 days per week during working hours, and all physicians were advised of this availability. In addition, patients presenting to the emergency room were also scanned at the discretion of the emergency physician. There were no exclusion criteria and US findings were recorded as part of the patient record.

### Ultrasound equipment

A portable LOGIQe US unit was provided by the General Electric Corporation (GE Medical, Milwaukee, WI, USA). The unit was equipped with a 2.5-MHz curvilinear probe, a 5-MHz phased array cardiac probe, a 7-MHz linear probe, and a 4.5-MHz endovaginal probe. There were Doppler and color flow capabilities in addition to software packages for all applications.

### Data collection

US images were acquired by one of two residency trained emergency physicians as part of routine patient care, and under consultation. In addition, there were scheduled US examination date/times that were available for obstetrical and medical inpatients. Patient demographics, presenting complaint and significant exam findings were recorded. Pre-US diagnosis was made by the treating physician. Ultrasound Images were recorded and digitally stored using still images and CINE loops. Upon completion of the study, a determination was made as to whether the US data changed patient management. All images were reviewed by an ARDMS certified sonographer with fellowship training in emergency US (CM) and assessed for accuracy upon return to the United States.

### Definitions

Pre-US diagnosis was made by the treating physician and post-US diagnosis was made upon completion of the US. Patients who met one or more of the following criteria were considered to have a change in management:

1. Differing pre-/post-US diagnosis.
2. Change in disposition after ultrasonography.
3. Referral for surgical intervention.
4. Addition or withdraw of pharmacotherapy.

Study types were defined by indication/anatomic modality [Table 1].

**Table 1:** Definition of study type

Study type	Definition
Pelvic	Pelvic US examination of the uterus and overies in patients known or presumed to be HCG negative
Second/third trimester pregnancy	Fetal and uterine examination of patients known or deemed to be in their second/third trimester of pregnancy
First trimester pregnancy	Fetal, Uterine, and ovarian examination of patients known or deemed to be in their first trimester of pregnancy
RUQ	Right upper quadrant US, carried out for suspected hapatobiliary disease
IP fluid	Intraperitoneal fluid analysis carried out for nontraumatic evaluation of ascites or abdominal distention
ECHO	Echo-cardiographic evaluation of the heart, nontraumatic
FAST	Focused assessment for sonography in trauma
Renal/bladder	Reanal and bladder US
Interventional	Interventional US for line placement, drainage of fluid/ascess collection, and fracture reduction
IT fluid	Evaluation of nontraumatic intrathoracic fluid collection. Noninterventional
Vascular	Vascular US for evaluation of arterial and venus aneurysm, thrombosis and anatomy. Noninterventional
Other	All other indications and use of US imaging.

## RESULTS

**Figure 1:**
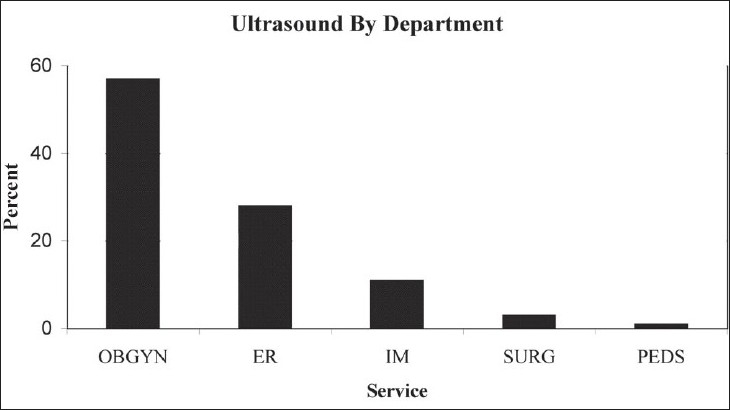
Percentage of studies carried out per inpatient department

A total of 126 examinations were performed on 102 consecutive patients enrolled in the study during a 5-week period. Eighty percent of enrolled patients were female, 20% male with an average age of 33 years (range 0-84). The majority of imaging was carried out in the obstetrical and emergency department settings [Figure 1].

**Figure 2:**
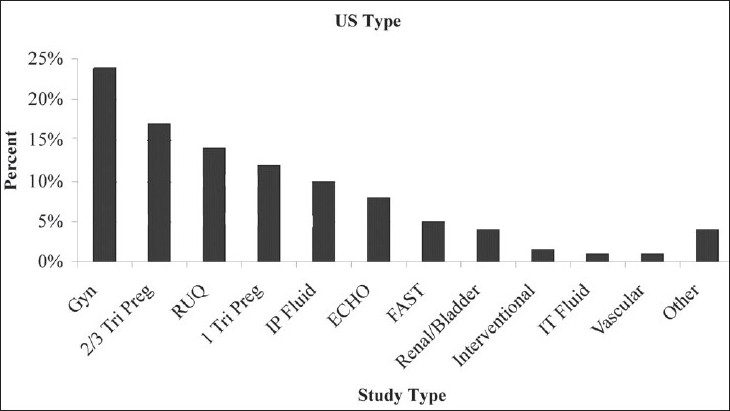
Percent distribution of imaging modality/type performed

Eighty percent of patients had one type of US performed, 15% had two study types, and 5% received three or more study modalities. The distribution of imaging modality is represented in Figure 2, with obstetrical and gynecological imaging studies as the predominant study type.

**Figure 3:**
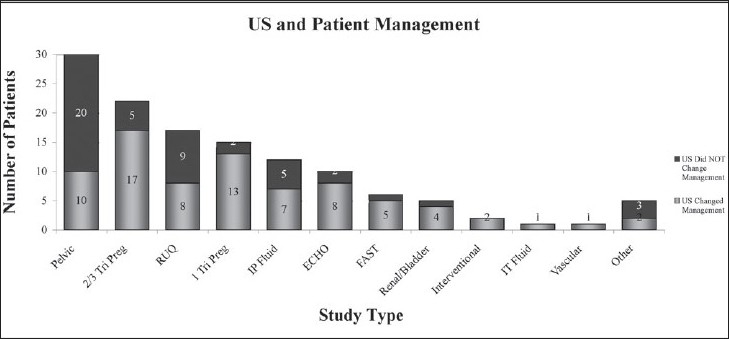
Ultrasound and patient management. Imaging modality and total cases for which US data changed patient management

Ultrasound investigations were deemed to have changed patient management in 62% of cases. This impact was greatest in cases of pregnancy, echocardiography, and focused assessment of sonography in trauma (FAST) examination [Figure 3, Table 2]. There was limited utility in patients receiving imaging for right upper quadrant (RUQ) and pelvic modalities. Over 80% of US studies were completed using the curvilinear probe. Follow-up data were limited as many patients were managed conservatively without operative intervention and confirmatory studies were not available. Pathologic confirmation was also not available. Review of ultrasounds by an attending emergency physician with fellowship training in ultrasound and American Registry of Diagnostic Medical Sonographer (ARDMS) certification did not reveal any significant differences in diagnoses.

**Table 2:** Study type and percentage of cases in which management was changed based on ultrasound data

Study type	*N*(%)	Changed management(%)
First trimester pregnancy	15(12)	86
FAST-trauma	6(5)	83
ECHO	10(8)	80
Second/third trimester pregnancy	22(18)	77
IP fluid	12(10)	58
RUQ	17(14)	47
Other	5(4)	40
Pelvic US	30(24)	33

## DISCUSSION

In 1984, a WHO scientific group on the Future Use of New Imaging Technologies in developing countries met in Geneva to consider the use of US in resource limited settings. As part of their recommendations, specifications for US systems (WHO-BRS) and indications for use were prescribed.[11] Furthermore, the WHO stated that in comparison to CT, MRI, and X-ray, the diagnostic problems for which US is particularly suited are much closer to the need of developing countries. Therefore, the acquisition of this equipment should have a higher priority in such countries and all efforts to promote its use and the necessary specialized training for physicians should be encouraged.[11]

Over the past 20 years, significant advancements have been generated in computer technology, and development of portable US technology has paralleled. Despite these advancements, limited progress on the introduction of US technology for low-income countries has been made. The use of CT is still prohibitively expensive in developing countries, as the cost of a single CT scanner would equal the entire year's budget of a tertiary referral hospital, such as JFK in Liberia. As such US can and should be used wherever applicable, as the first and often only examination, with the intention of providing a definitive or at least a focused differential diagnosis.[2]

### Utility of US in patient management

In this investigation, US was deemed to have changed patient management in 62% of cases. The methodology used in determining a “change in management” may be criticized as being subjective and likely introduces a certain degree of observer bias. Interestingly however, this figure has been replicated consistently in the literature using similar methodology both in the United States and abroad.

In a United States survey of the perceived efficacy of sonography in making diagnostic and management decisions, 68% of referring physicians considered sonography valuable or most valuable for patient management.[13] Prospective data from Rwanda in 227 patients showed that 60.8% of scans were abnormal and that findings contributed to the resolution of the problem in 71% of these patients.[14] Data from Cameroon suggest that US was contributive in 62% of patients, and a similar study from Sudan found that US confirmed or excluded the diagnosis in 69% of patients imaged.[3,10] Finally, data from portable echocardiographic studies carried out in Gambia and Mexico showed that US helped to clarify the clinical problem in 65% and 63% of cases, respectively.[4,5] These data, and ours, support the conclusion that the introduction of US technology has the potential to change management in approximately two-thirds of patients who receive US imaging.

In the context of a gross assessment of the utility of US in resource-constrained environments, we attempted to further stratify the impact US had on patient management. This was done based on the study type utilized as relevant to the indication for imaging. The present data [Figure 3] suggest that imaging carried out for first trimester pregnancy, FAST evaluation, echocardiography, and second/third trimester pregnancy had the greatest impact on patient management, with 77-86% of patients having a change in management as a result of information gained from the US [Table 2]. Pelvic and RUQ sonography was only useful in 33% and 47%, respectively.

### Obstetrical application

**Figure 4:**
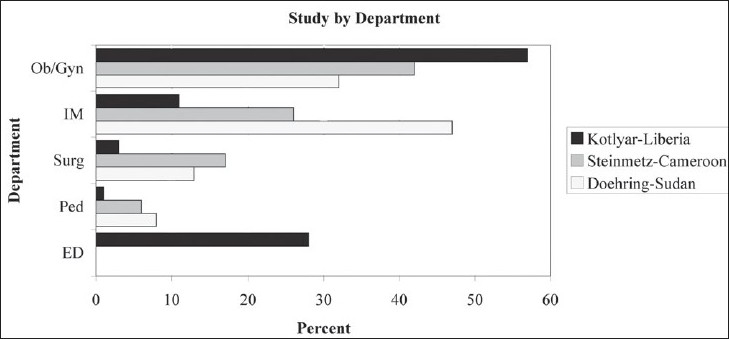
Percentage of US carried out by department. Comparison of data from Liberia, Cameroon, and Sudan[3,10]

In our series, 80% of enrolled patients were female. This large proportion of female patients is reflective of the fact that 57% of patients were referred from the department of obstetrics and gynecology. Eleven percent of patients were medical, 3% surgical, and only 1% pediatric. Twenty-eight percent of patients were enrolled in the emergency department. The population of patients is representative of all patients treated at this hospital, and its departmental referral is consistent with those seen in similar studies carried out in Cameroon and the Sudan [Figure 4].[3,10] Indications for US examination paralleled this distribution [Figure 2].

In this series and others, the largest number of patients referred for US came from the department of obstetrics and gynecology.[3,10] Furthermore, the greatest impact on changes in patient management was seen in the context of obstetrical care. These findings suggest that the primary focus for the introduction and programming of US technology and training in developing countries should target the delivery of US in obstetrical care. This is consistent with recommendations put forth by the WHO and should continue to be the primary effort for the introduction and development of US in areas that currently do not have access to the technology.[11]

In our series, the main indications for obstetrical US (data not presented) were for evaluation of: first trimester vaginal bleeding/abdominal pain; fetal demise; estimation of gestational age; multiple gestation; and emergent care of suspected placenta previa and abruption. These indications are consistent with WHO guidelines and service the majority of essential maternal care.[11] Education and training should be focused on key stakeholders in these departments and should extend to physicians and mid-level providers. In this setting, the provision of US imaging for the care of obstetrical patients is considered essential.[11,14,15]

### Other applications

Beyond application in obstetrical disease, US in developing countries will often serve as the first and only diagnostic examination a patient may receive, and as such earlier and more comprehensive diagnostic evaluation would be warranted.[2] In this series, 28% of patients were imaged in the ED and significant changes in management strategies were made based on echocardiography, FAST examination, and abdominal US [Table 2]. Other studies have shown similar impact on diagnosis and treatment of abdominal pain while the utility of US in management of trauma in developing countries has yet to be reported.[3,6,13,14] We recommend a secondary focus for US training in developing countries as relevant to its implementation in the delivery of emergency care services.

### Equipment

Over 80% of studies in this series were carried out with a 2.5-MHz curvilinear probe. Given the resource constraints, we recommend that all units be standardized with this transducer. Optional units include endovaginal transducers, phased array cardiac probes, and linear transducers as relevant and available. In settings where electrical supply and patient transport issues are of concern, we support the use of portable US devices with battery power and rugged construction. Although years of operation without technical difficulty have been reported, failure and breakdown should be anticipated and provisions for repair and maintenance are essential.[10]

### Future prospectus

This study opens the potential for further investigation into specific outcome metrics as relevant to perinatal care. Future studies will focus on measurement of gross indicators of utility as relevant to perinatal mortality. Additional investigations will address issues of US education and training and the feasibility and requirements for successful implementation. Given our initial assessment which identifies obstetrical US as the primary utility for US development in Liberia, we hope to develop a methodology and protocol for the education and training of US in this setting. We plan to provide curricula and training modules for rapid and successful implementation with concomitant evaluation of impact data.

## CONCLUSIONS

A primary role for US in developing countries is in the diagnosis and treatment of obstetrical disease, with a secondary role for diagnosis of traumatic and a traumatic intra-abdominal processes. Most needs can be met with the curvilinear probe. Training should begin with obstetrical care and should be a primary focus for curriculum in developing nations.
